# 4-Dimethyl­amino-*N*′-(2-hy­droxy-3,5-diiodo­benzyl­idene)benzohydrazide

**DOI:** 10.1107/S1600536811035070

**Published:** 2011-09-03

**Authors:** Fu-Lin Mao, Wen-Sheng Li, Xiao-Ping Zhou

**Affiliations:** a College of Chemistry and Chemical Engineering, Hunan University, Changsha 410082, People’s Republic of China; b Department of Chemistry, Yancheng Normal College, Yancheng 224002, People’s Republic of China

## Abstract

The title mol­ecule, C_16_H_15_I_2_N_3_O_2_, adopts an *E* configuration about the C=N bond. The dihedral angle between the two benzene rings is 6.4 (2)°. An intra­molecular O—H⋯N hydrogen bond occurs. In the crystal, mol­ecules are linked through inter­molecular N—H⋯O hydrogen bonds, forming chains propagating in the *c*-axis direction.

## Related literature

For medical applications of hydrazones, see: Ajani *et al.* (2010 ▶); Zhang *et al.* (2010 ▶); Angelusiu *et al.* (2010 ▶). For related structures, see: Su *et al.* (2011*a* ▶,*b* ▶); Khaledi *et al.* (2010 ▶); Zhou & Yang (2010 ▶); Ji & Lu (2010 ▶); Singh & Singh (2010 ▶); Ahmad *et al.* (2010 ▶). For similar compounds that we have reported recently, see: Dai & Mao (2010*a* ▶,*b* ▶).
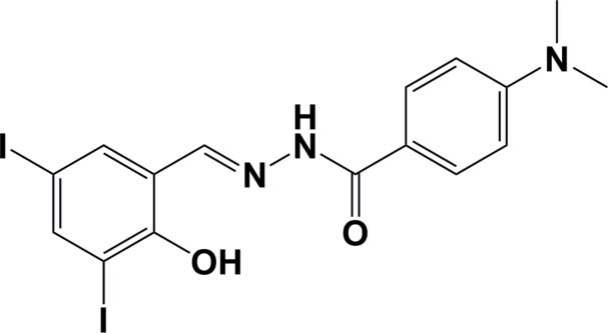


## Experimental

### 

#### Crystal data

C_16_H_15_I_2_N_3_O_2_
*M* *_r_* = 535.11
Monoclinic, 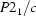

*a* = 20.387 (4) Å
*b* = 9.0000 (16) Å
*c* = 9.8355 (17) Å
β = 94.320 (2)°
*V* = 1799.5 (5) Å^3^
*Z* = 4
Mo *K*α radiation
μ = 3.51 mm^−1^
*T* = 298 K
0.17 × 0.17 × 0.15 mm

#### Data collection

Bruker SMART CCD area-detector diffractometer
Absorption correction: multi-scan (*SADABS*; Bruker, 2001 ▶) *T*_min_ = 0.587, *T*_max_ = 0.621
9471 measured reflections
3892 independent reflections
2551 reflections with *I* > 2σ(*I*)
*R*_int_ = 0.033

#### Refinement

*R*[*F* ^2^ > 2σ(*F* ^2^)] = 0.042
*wR*(*F* ^2^) = 0.094
*S* = 1.04
3892 reflections
214 parameters
1 restraint
H atoms treated by a mixture of independent and constrained refinement
Δρ_max_ = 0.70 e Å^−3^
Δρ_min_ = −1.06 e Å^−3^

### 

Data collection: *SMART* (Bruker, 2007 ▶); cell refinement: *SAINT* (Bruker, 2007 ▶); data reduction: *SAINT*; program(s) used to solve structure: *SHELXS97* (Sheldrick, 2008 ▶); program(s) used to refine structure: *SHELXL97* (Sheldrick, 2008 ▶); molecular graphics: *SHELXTL* (Sheldrick, 2008 ▶); software used to prepare material for publication: *SHELXTL*.

## Supplementary Material

Crystal structure: contains datablock(s) global, I. DOI: 10.1107/S1600536811035070/su2309sup1.cif

Structure factors: contains datablock(s) I. DOI: 10.1107/S1600536811035070/su2309Isup2.hkl

Supplementary material file. DOI: 10.1107/S1600536811035070/su2309Isup3.cml

Additional supplementary materials:  crystallographic information; 3D view; checkCIF report

## Figures and Tables

**Table 1:** Hydrogen-bond geometry (Å, °)

*D*—H⋯*A*	*D*—H	H⋯*A*	*D*⋯*A*	*D*—H⋯*A*
O1—H1⋯N1	0.82	1.91	2.623 (5)	144
N2—H2⋯O2^i^	0.90 (1)	2.16 (2)	3.016 (5)	159 (5)

Symmetry code: (i) 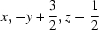.

**Fig. 1.**
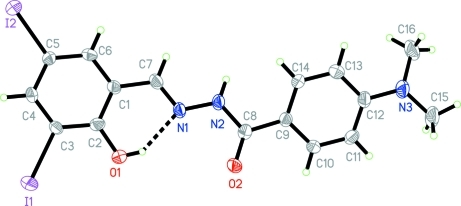
The molecular structure of the title molecule, showing 30% probability displacement ellipsoids and the atomic numbering. The intramolecular N-H···O hydrogen bond is shown as a dashed line (See Table 1 for details).

**Fig. 2.**
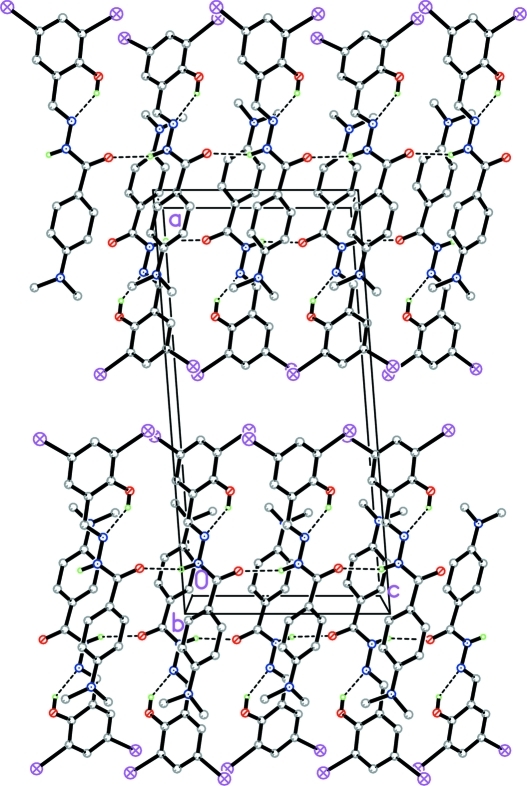
Crystal packing of the title compound, viewed down the b axis, with the O-H···N and N-H···O hydrogen bonds shown as dashed lines (see Table 1 for details).

## supplementary crystallographic information

### Comment

In the last few years, medical applications of a number of hydrazone compounds have received considerable attention (Ajani *et al.*, 2010; Zhang *et al.*, 2010; Angelusiu *et al.*, 2010). The structures of several hydrazone derivatives have also been determined (Su *et al.*, 2011*a*,*b*; Khaledi *et al.*, 2010; Zhou & Yang, 2010; Ji & Lu, 2010; Singh & Singh, 2010; Ahmad *et al.*, 2010). As a continuation of our work in this area (Dai & Mao, 2010*a*,*b*), we report herein on the structure of the new title hydrazone compound.

In the molecule of the title compound, there is an intramolecular O—H···N hydrogen bond, as shown in Fig. 1. The dihedral angle between the (C1-C6) and (C9-C14) benzene rings is 6.4 (2)°. The bond lengths and angles are comparable to those found in the hydrazone compounds cited above.

In the crystal, the hydrazone molecules are linked through intermolecular N—H···O hydrogen bonds (Table 1), to form one-dimensional chains in the *c* direction (Fig. 2).

### Experimental

The reaction of 2-hydroxy-3,5-diiodobenzaldehyde (0.374 g, 1 mmol) with 4-dimethylaminobenzohydrazide (0.179 g, 1 mmol) in 50 ml methanol at room temperature afforded the title compound. Colorless block-shaped single crystals were formed by slow evaporation of the clear solution in air.

### Refinement

The H2 atom was located in a difference Fourier map and refined with a distance restraint, N—H = 0.90 (1) Å, and *U*_iso_ = 0.08 Å^2^. The other H-atoms were positioned geometrically and refined as riding: O—H = 0.82 Å, C—H = 0.93 and 0.96 Å, for CH and CH_3_ H-atoms, respectively, with *U*_iso_(H) = k × *U*_eq_(O,C), where k = 1.5 for OH and CH_3_ H-atoms and k = 1.2 for all other H-atoms.

### Crystal data

**Table:**

C_16_H_15_I_2_N_3_O_2_	*F*(000) = 1016
*M**_r_* = 535.11	*D*_x_ = 1.975 Mg m^−^^3^
Monoclinic, *P*2_1_/*c*	Mo *K*α radiation, λ = 0.71073 Å
Hall symbol: -P 2ybc	Cell parameters from 2040 reflections
*a* = 20.387 (4) Å	θ = 2.4–24.5°
*b* = 9.0000 (16) Å	µ = 3.51 mm^−^^1^
*c* = 9.8355 (17) Å	*T* = 298 K
β = 94.320 (2)°	Block, colourless
*V* = 1799.5 (5) Å^3^	0.17 × 0.17 × 0.15 mm
*Z* = 4	

### Data collection

**Table:**

Bruker SMART CCD area-detector diffractometer	3892 independent reflections
Radiation source: fine-focus sealed tube	2551 reflections with *I* > 2σ(*I*)
graphite	*R*_int_ = 0.033
ω scans	θ_max_ = 27.0°, θ_min_ = 2.5°
Absorption correction: multi-scan (*SADABS*; Bruker, 2001)	*h* = −25→18
*T*_min_ = 0.587, *T*_max_ = 0.621	*k* = −11→11
9471 measured reflections	*l* = −12→12

### Refinement

**Table:**

Refinement on *F*^2^	Primary atom site location: structure-invariant direct methods
Least-squares matrix: full	Secondary atom site location: difference Fourier map
*R*[*F*^2^ > 2σ(*F*^2^)] = 0.042	Hydrogen site location: inferred from neighbouring sites
*wR*(*F*^2^) = 0.094	H atoms treated by a mixture of independent and constrained refinement
*S* = 1.04	*w* = 1/[σ^2^(*F*_o_^2^) + (0.036*P*)^2^ + 0.0912*P*] where *P* = (*F*_o_^2^ + 2*F*_c_^2^)/3
3892 reflections	(Δ/σ)_max_ < 0.001
214 parameters	Δρ_max_ = 0.70 e Å^−^^3^
1 restraint	Δρ_min_ = −1.06 e Å^−^^3^

### Special details

**Table:**

Geometry. All esds (except the esd in the dihedral angle between two l.s. planes) are estimated using the full covariance matrix. The cell esds are taken into account individually in the estimation of esds in distances, angles and torsion angles; correlations between esds in cell parameters are only used when they are defined by crystal symmetry. An approximate (isotropic) treatment of cell esds is used for estimating esds involving l.s. planes.
Refinement. Refinement of F^2^ against ALL reflections. The weighted R-factor wR and goodness of fit S are based on F^2^, conventional R-factors R are based on F, with F set to zero for negative F^2^. The threshold expression of F^2^ > 2sigma(F^2^) is used only for calculating R-factors(gt) etc. and is not relevant to the choice of reflections for refinement. R-factors based on F^2^ are statistically about twice as large as those based on F, and R- factors based on ALL data will be even larger.

### Fractional atomic coordinates and isotropic or equivalent isotropic displacement parameters (Å^2^)

**Table:**

	*x*	*y*	*z*	*U*_iso_*/*U*_eq_	
I1	0.430896 (19)	0.68628 (4)	0.37673 (4)	0.06296 (15)	
I2	0.41396 (2)	0.29856 (5)	−0.12108 (4)	0.07864 (18)	
N1	0.17425 (19)	0.6984 (4)	0.1129 (4)	0.0460 (10)	
N2	0.1097 (2)	0.7279 (5)	0.0734 (4)	0.0486 (11)	
N3	−0.1967 (2)	0.8856 (6)	−0.0186 (5)	0.0745 (15)	
O1	0.28501 (17)	0.7467 (4)	0.2597 (3)	0.0556 (9)	
H1	0.2449	0.7485	0.2442	0.083*	
O2	0.09491 (16)	0.8441 (4)	0.2731 (3)	0.0528 (9)	
C1	0.2749 (2)	0.5829 (5)	0.0659 (4)	0.0411 (12)	
C2	0.3110 (2)	0.6455 (5)	0.1782 (5)	0.0423 (12)	
C3	0.3763 (2)	0.6018 (5)	0.2055 (5)	0.0400 (11)	
C4	0.4057 (2)	0.5002 (5)	0.1236 (4)	0.0407 (11)	
H4	0.4491	0.4712	0.1439	0.049*	
C5	0.3697 (2)	0.4431 (5)	0.0118 (5)	0.0408 (11)	
C6	0.3058 (2)	0.4842 (5)	−0.0169 (5)	0.0459 (12)	
H6	0.2824	0.4452	−0.0936	0.055*	
C7	0.2062 (2)	0.6198 (6)	0.0322 (5)	0.0481 (13)	
H7	0.1853	0.5858	−0.0492	0.058*	
C8	0.0719 (2)	0.8033 (5)	0.1611 (5)	0.0427 (12)	
C9	0.0026 (2)	0.8230 (5)	0.1103 (5)	0.0406 (11)	
C10	−0.0353 (2)	0.9280 (5)	0.1713 (5)	0.0457 (13)	
H10	−0.0161	0.9855	0.2423	0.055*	
C11	−0.1005 (2)	0.9493 (6)	0.1295 (5)	0.0480 (13)	
H11	−0.1245	1.0206	0.1731	0.058*	
C12	−0.1311 (2)	0.8675 (6)	0.0245 (5)	0.0500 (13)	
C13	−0.0930 (3)	0.7631 (7)	−0.0376 (6)	0.0675 (17)	
H13	−0.1121	0.7059	−0.1088	0.081*	
C14	−0.0279 (3)	0.7425 (6)	0.0035 (5)	0.0563 (14)	
H14	−0.0037	0.6728	−0.0414	0.068*	
C15	−0.2392 (3)	0.9775 (7)	0.0537 (6)	0.0759 (19)	
H15A	−0.2365	0.9488	0.1480	0.114*	
H15B	−0.2837	0.9659	0.0155	0.114*	
H15C	−0.2261	1.0794	0.0464	0.114*	
C16	−0.2223 (3)	0.8274 (7)	−0.1472 (6)	0.083 (2)	
H16A	−0.1876	0.8203	−0.2076	0.124*	
H16B	−0.2561	0.8922	−0.1862	0.124*	
H16C	−0.2405	0.7305	−0.1341	0.124*	
H2	0.095 (3)	0.714 (6)	−0.014 (2)	0.080*	

### Atomic displacement parameters (Å^2^)

**Table:**

	*U*^11^	*U*^22^	*U*^33^	*U*^12^	*U*^13^	*U*^23^
I1	0.0550 (3)	0.0776 (3)	0.0542 (3)	−0.0025 (2)	−0.00991 (18)	−0.02124 (19)
I2	0.0613 (3)	0.0960 (4)	0.0767 (3)	0.0256 (2)	−0.0072 (2)	−0.0429 (2)
N1	0.034 (2)	0.068 (3)	0.036 (2)	0.010 (2)	0.0000 (18)	0.004 (2)
N2	0.036 (2)	0.075 (3)	0.034 (2)	0.015 (2)	0.0013 (19)	0.003 (2)
N3	0.041 (3)	0.121 (4)	0.060 (3)	0.024 (3)	−0.007 (2)	−0.013 (3)
O1	0.049 (2)	0.071 (2)	0.046 (2)	0.011 (2)	0.0005 (19)	−0.0170 (18)
O2	0.044 (2)	0.083 (3)	0.0309 (19)	0.0048 (18)	−0.0020 (16)	−0.0016 (18)
C1	0.040 (3)	0.055 (3)	0.028 (3)	0.004 (2)	0.000 (2)	0.002 (2)
C2	0.051 (3)	0.045 (3)	0.032 (3)	0.002 (2)	0.006 (2)	−0.002 (2)
C3	0.036 (3)	0.047 (3)	0.036 (3)	−0.003 (2)	−0.004 (2)	−0.003 (2)
C4	0.034 (3)	0.045 (3)	0.042 (3)	0.006 (2)	−0.003 (2)	0.007 (2)
C5	0.041 (3)	0.046 (3)	0.036 (3)	0.007 (2)	0.004 (2)	−0.004 (2)
C6	0.049 (3)	0.054 (3)	0.034 (3)	−0.002 (3)	−0.003 (2)	−0.009 (2)
C7	0.040 (3)	0.065 (3)	0.038 (3)	0.004 (3)	−0.006 (2)	0.002 (3)
C8	0.044 (3)	0.050 (3)	0.035 (3)	0.007 (2)	0.007 (2)	0.011 (2)
C9	0.036 (3)	0.051 (3)	0.034 (3)	0.006 (2)	0.002 (2)	0.005 (2)
C10	0.043 (3)	0.057 (3)	0.036 (3)	0.004 (3)	−0.002 (2)	−0.004 (2)
C11	0.050 (3)	0.056 (3)	0.039 (3)	0.014 (3)	0.008 (2)	−0.001 (2)
C12	0.037 (3)	0.068 (4)	0.045 (3)	0.008 (3)	0.004 (2)	0.006 (3)
C13	0.051 (4)	0.099 (5)	0.051 (4)	0.008 (3)	−0.008 (3)	−0.025 (3)
C14	0.040 (3)	0.074 (4)	0.054 (3)	0.012 (3)	0.000 (3)	−0.022 (3)
C15	0.043 (3)	0.099 (5)	0.085 (5)	0.023 (3)	0.004 (3)	0.008 (4)
C16	0.046 (4)	0.139 (6)	0.060 (4)	−0.001 (4)	−0.014 (3)	0.001 (4)

### Geometric parameters (Å, °)

**Table:**

I1—C3	2.091 (4)	C6—H6	0.9300
I2—C5	2.096 (4)	C7—H7	0.9300
N1—C7	1.278 (6)	C8—C9	1.474 (6)
N1—N2	1.371 (5)	C9—C14	1.385 (6)
N2—C8	1.378 (6)	C9—C10	1.385 (6)
N2—H2	0.898 (10)	C10—C11	1.375 (6)
N3—C12	1.380 (6)	C10—H10	0.9300
N3—C15	1.427 (7)	C11—C12	1.380 (7)
N3—C16	1.431 (7)	C11—H11	0.9300
O1—C2	1.348 (5)	C12—C13	1.390 (7)
O1—H1	0.8200	C13—C14	1.370 (7)
O2—C8	1.221 (5)	C13—H13	0.9300
C1—C6	1.389 (6)	C14—H14	0.9300
C1—C2	1.400 (6)	C15—H15A	0.9600
C1—C7	1.452 (6)	C15—H15B	0.9600
C2—C3	1.393 (6)	C15—H15C	0.9600
C3—C4	1.385 (6)	C16—H16A	0.9600
C4—C5	1.375 (6)	C16—H16B	0.9600
C4—H4	0.9300	C16—H16C	0.9600
C5—C6	1.363 (6)		
			
C7—N1—N2	117.1 (4)	N2—C8—C9	114.4 (4)
N1—N2—C8	119.2 (4)	C14—C9—C10	117.0 (4)
N1—N2—H2	120 (4)	C14—C9—C8	123.9 (4)
C8—N2—H2	120 (4)	C10—C9—C8	119.1 (4)
C12—N3—C15	121.7 (5)	C11—C10—C9	121.5 (5)
C12—N3—C16	120.6 (5)	C11—C10—H10	119.2
C15—N3—C16	117.2 (5)	C9—C10—H10	119.2
C2—O1—H1	109.5	C10—C11—C12	121.5 (5)
C6—C1—C2	119.0 (4)	C10—C11—H11	119.2
C6—C1—C7	119.0 (4)	C12—C11—H11	119.2
C2—C1—C7	122.0 (4)	C11—C12—N3	122.7 (5)
O1—C2—C3	119.3 (4)	C11—C12—C13	117.0 (5)
O1—C2—C1	122.3 (4)	N3—C12—C13	120.3 (5)
C3—C2—C1	118.5 (4)	C14—C13—C12	121.5 (5)
C4—C3—C2	121.6 (4)	C14—C13—H13	119.2
C4—C3—I1	118.7 (3)	C12—C13—H13	119.2
C2—C3—I1	119.7 (3)	C13—C14—C9	121.5 (5)
C5—C4—C3	118.9 (4)	C13—C14—H14	119.3
C5—C4—H4	120.6	C9—C14—H14	119.3
C3—C4—H4	120.6	N3—C15—H15A	109.5
C6—C5—C4	120.6 (4)	N3—C15—H15B	109.5
C6—C5—I2	119.2 (3)	H15A—C15—H15B	109.5
C4—C5—I2	120.1 (3)	N3—C15—H15C	109.5
C5—C6—C1	121.4 (4)	H15A—C15—H15C	109.5
C5—C6—H6	119.3	H15B—C15—H15C	109.5
C1—C6—H6	119.3	N3—C16—H16A	109.5
N1—C7—C1	120.8 (4)	N3—C16—H16B	109.5
N1—C7—H7	119.6	H16A—C16—H16B	109.5
C1—C7—H7	119.6	N3—C16—H16C	109.5
O2—C8—N2	121.2 (4)	H16A—C16—H16C	109.5
O2—C8—C9	124.3 (4)	H16B—C16—H16C	109.5

### Hydrogen-bond geometry (Å, °)

**Table:**

*D*—H···*A*	*D*—H	H···*A*	*D*···*A*	*D*—H···*A*
O1—H1···N1	0.82	1.91	2.623 (5)	144.
N2—H2···O2^i^	0.90 (1)	2.16 (2)	3.016 (5)	159 (5)

Symmetry codes: (i) *x*, −*y*+3/2, *z*−1/2.
